# Experience of Online Learning from COVID-19: Preparing for the Future of Digital Transformation in Education

**DOI:** 10.3390/ijerph192416787

**Published:** 2022-12-14

**Authors:** Bo Jiang, Xinya Li, Sijiang Liu, Chuanyan Hao, Gangyao Zhang, Qiaomin Lin

**Affiliations:** School of Education Science and Technology, Nanjing University of Posts and Telecommunications, Nanjing 210023, China

**Keywords:** post-pandemic era, digital learning tools, COVID-19, health care

## Abstract

COVID-19 has affected traditional instructional activities. Home-based isolation and restrictive movement measures have forced most learning activities to move from an offline to an online environment. Multiple studies have also demonstrated that teaching with virtual tools during the COVID-19 pandemic is always ineffective. This study examines the different characteristics and challenges that virtual tools brought to online education in the pre-pandemic and pandemic era, with the aim of providing experience of how virtual tools supported purely online learning during a health crisis. By searching keywords in public databases and review publications, this study tries to summarize the major topics related to the research theme. These topics are the characteristics of learning supported by technologies in pre-pandemic and pandemic era, the challenges that education systems have faced during the COVID-19 pandemic. This study also compares the functions, advantages and limitations of typical virtual tools, which has rarely been done in previous studies. This study tries to present the features of virtual tools that support online learning and the challenges regarding real-life risk scenarios, and tries to provide educational institutions with a distinct perspective for efficient teaching and learning in future potential health crises.

## 1. Introduction

**Figure 1:**
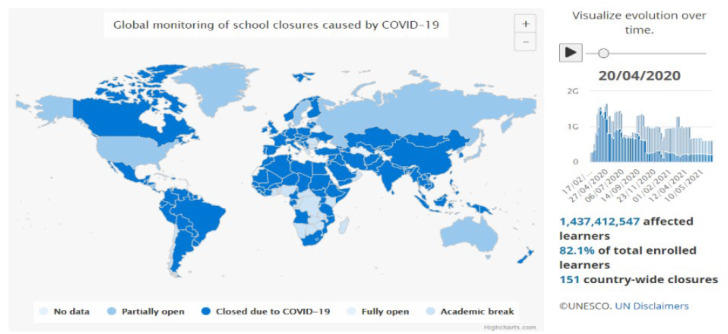
Global monitoring of school lockdowns affected by the COVID-19 pandemic (UNESCO report) [10].

Health crises refer to a series of negative consequences caused by unexpected events such as infectious disease or natural disaster [1]. The outbreak of the SARS virus in mainland China in 2003 and the outbreak of Ebola in Africa in 2013 mainly affected people’s life during a specific period [2,3]. Almost a decade later, a new coronavirus called Severe Acute Respiratory Syndrome Coronavirus 2 (SARS-CoV-2) spread all over the world and caused irreversible harm to both finances and people’s lives [4]. To prevent the rapid spread of the virus, home-based isolation is the primary measure to interrupt the spread of the pandemic [5,6]. In the education sector, governments decided to close local schools, and the education system was briefly suspended [7,8,9]. Compulsory school closures have impacted approximately 151 countries and 1,437,412,547 students during the COVID-19 pandemic, as of 20 April 2020 (Figure 1) [10]. Internet-based learning with a variety of virtual tools appears to be the only method of secure learning.

The COVID-19 pandemic had not occurred when online learning first appeared. Whether it was broadcast, television, or print media in the early days, all have transformed or enriched traditional education [11]. With the growth of information and communication technology (ICT), online resource platforms, learning-management systems, instant-messaging apps, and other digital technologies have improved conventional formal and informal education in the past decade [12,13,14,15]. These innovations assist in transforming the student’s position from information recipient to knowledge creator and broadcaster [16].

Due to home-based isolation limits, most schools found alternative digital technologies for online instruction [17]. Digital technologies help students study, communicate, and exchange learning materials [18]. During the pandemic, most virtual tools helped to create a virtual learning environment [19]. However, online education did not function as intended. Many students said that using synchronous meeting technologies during the pandemic even disturbed them [20]. Teachers were also afraid that students would have trouble connecting with them, and that it would be challenging to evaluate their learning progress in online learning [21]. Unpredictable network disconnections have become another obstacle for students and teachers who want to complete their education online [22]. Thus, some researchers have proposed that virtual tools have limits in applications in education, and students should return to face-to-face classes after the pandemic. For example, Neuwirth et al. contend that learning online with digital resources is the most reasonable course of action during a public health emergency. However, when students return to their regular routines, they should continue to study in a face-to-face classroom [23].

It is undeniable that COVID-19 provides an excellent opportunity to reconsider how virtual technologies and online learning practices may promote learning. The outbreak of COVID-19 has pushed people to rethink the various issues of online learning. This study aims to give a more in-depth examination of the difficulties and solutions inherent in virtual-tool-assisted education in the pre-pandemic era and during health crises. It provides new insights and a wider direction for education in the post-pandemic era by illustrating the shifting tendencies connected with these problems. The roles, functions, and moral implications of numerous virtual tools supporting online learning in COVID-19 have been investigated in recent studies [24,25,26,27]. This study used a longitudinal method to classify the investigated results into pre-pandemic and pandemic onset. This study also compared the strengths and weaknesses of specific digital tools in their application. We present the results in this manner because the pandemic occurred suddenly and with specificity, and it is more appropriate to demonstrate the different applications and features of virtual tools in education over time.

Thus, these research objects (ROs) can be summarized in three points:

- RO1: Analysis and summary of the characteristics of virtual-tool-supported learning in the pre-pandemic and pandemic era.
- RO2: A cross-sectional comparison of virtual-tool functions, benefits, and limits.
- RO3: Discovery of the challenges facing purely online learning during the COVID-19 pandemic.

The purpose of this series of examinations and analyses is to prepare for the next step, which is to start from existing experiences and find how digital tools can be more effectively integrated into educational applications. In contrast to just supporting normal teaching and learning services, this will improve education quality in particular contexts of unforeseeable public health risks. In addition to the above investigation, this research seeks to answer the following three key questions:

- RQ1: What were the main virtual-tool characteristics, and how did they support traditional learning before COVID-19?
- RQ2: What characteristics of the main virtual tools supported online learning during COVID-19?
- RQ3: What were the main challenges of virtual-tool-based learning during and before COVID-19?

The answers to each question are arranged in Section 3, Section 4 and Section 5, respectively. This study summarizes the existing experiences of virtual tools that support education during the pre-pandemic pandemic eras, and to illustrate the challenges encountered by purely online learning practices during the pandemic. These findings may assist in finding ways of reducing the effects that health emergencies could have on learning. This study hopes to shed light on the role and functions of online learning in unforeseeable future global health crises.

## 2. Materials and Methods

**Figure 2:**
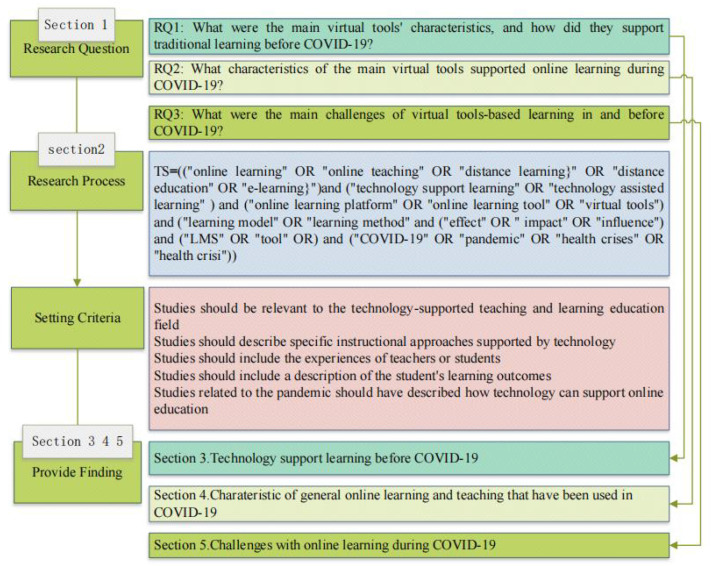
Critical steps of this review.

This study uses a systematic literature review approach to review the role of learning supported by virtual tools pre-pandemic and during the pandemic in prior literature. Based on this, we summarize the challenges of online learning assisted by virtual tools during the pandemic and explore the way that technology may assist the digital transformation of education in unforeseeable future learning scenarios, especially with regard to health risk. The systematic literature review is a rigorous and complex process, in which the results of prior research are critically examined and summarized as evidence to answer the research question [28]. This study was conducted strictly according to the criteria of Preferred Reporting Items for Systematic Reviews and Meta-analyses (PRISMA) [29]. The following diagram (Figure 2) represents several critical steps of this review. This review was not registered in the systematic review database. To ensure the timeliness of the included articles, we set the search period from 1 January 2008 to 1 April 2022. Researchers searched the literature on the Web of Science (WoS) core database. The keyword search follows the most common search technique, Boolean operators, to improve the effectiveness of the literature search [30].

We searched for research in the Web of Science (WOS) core database. Search key strings included online learning, online teaching, distance learning, distance education, e-learning, technology-assisted learning, online learning platforms, online learning tool, virtual tools, learning models, health crises, and COVID-19 etc. The search scope contains the title, abstract, or keywords. The specific search strings are represented in Table 1.

### 2.1. Criteria

Studies were examined based on the following criteria to ensure the high quality of the selected articles. First, only articles published in journals were included, while proceedings papers, book chapters, data papers, editorial materials, early access and reviews were excluded. Furthermore, the research included in this study must have been written in English. Studies not in English were excluded. All the above is to ensure the quality of the chosen journal. Finally, considering this study’s research theme and aims, the articles searched for in the dataset were further examined. The articles must describe the instructional features assisted by tools. Thus, the articles must describe how virtual tools might help offline or online teaching and learning. Table 2 and Table 3 list the specific inclusion and exclusion criteria.

### 2.2. Systematic Review Process

A literature review facilitates a retrospective approach to understanding the development process of a particular field [31]. However, to avoid subjectivity and implicit bias in the review process by researchers, it is essential to follow a rigorous procedure in the review process [32].

The researcher first conducted a literature search on the Web of Science using the search string, and 1857 articles were found. To ensure the quality of the included articles, proceedings papers, book chapters, data papers, editorial materials, early access, and reviews were excluded from the literature pool. After subtracting the open-access articles, only 737 articles entered the next screening stage. Based on the peer-review method used by Pahlevan et al., the researchers read the title, abstract, and keywords of the article to decide if the article should be included in the next screening stage [33]. After excluding the corresponding articles according to the criteria, 82 articles could proceed to the next review stage. A total of 655 articles were excluded because they did not meet the criteria.

**Figure 3:**
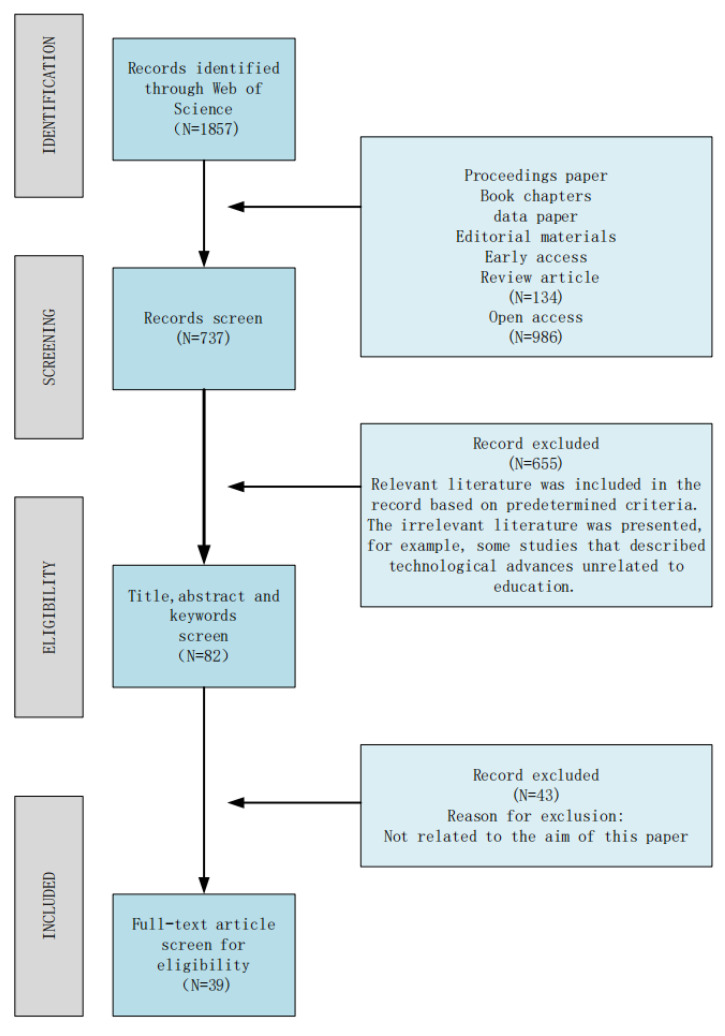
The flow chart of the whole review process.

Finally, 39 articles were included in the final criteria after the authors reached a consensus to read the full text. Figure 3 shows the flow chart of the whole review process.

## 3. Characteristic of General Online Learning and Teaching That before COVID-19

The development of information and communications technology and the emergence of the digital age has hastened the iteration of technology. The lightning-fast speed at which information can be processed and shared affects every part of life today. The education sector is also sensitive to fast technological changes [34]. Exploring appropriate ways to apply digital technology in face-to-face class (also called in-person class) is called digital transformation in the education era [35]. Hiltz and Turoff suggested that using digital technology in the classroom and turning traditional face-to-face learning into blended learning is the highest goal of the current digital transformation in education [36]. Many researchers are open-minded about using virtual tools in formal and informal learning environments [37,38]. However, it is not easy to use virtual technologies well in an actual learning environment. With the advancement of information and communications technology and the Internet, teachers are no longer satisfied with simply using technology in the traditional classroom. They are instead exploring new avenues for digital transformation in education [39,40].

Future directions of digital transformation are being explored in education as a result of advancement in information technology [39,40]. The rapid development of technology and its success in other areas has led most educators to become more positive about technology-enabled education [41,42]. In terms of faculty readiness to choose technology in teaching before the pandemic, most teachers preferred to follow traditional teaching strategies and maintain their old instructing habits when unfamiliar with virtual tools [43]. Fewer teachers were ready to abandon the face-to-face traditional learning scenario; some teachers knew how valuable virtual tools were in teaching.

Before the COVID-19 pandemic, most teachers in schools would recommend high-quality learning resources as pre-reading or for review outside the classroom. High-quality and free learning resources exist in massive open online courses (MOOCs), helping heterogeneous students learn at their own pace outside the classroom [44]. However, most MOOC platforms do not allow students and teachers to communicate in real time during the learning process. As a result, MOOC learning without real-time interaction may diminish student engagement [45].

Instant-messaging apps can also support long-distance contacts between students and teachers more easily than before COVID-19. Teachers are increasingly embracing instant-messaging apps such as WeChat and WhatsApp to interact with their students after class [46]. In particular, university teachers prefer to use WeChat to issue notifications and tasks in China. However, it is also controversial to use social media in class due to the absence of management of social media. Some researchers even argue that instant-messaging tools are too disruptive to apply in traditional classrooms. Abuse of these entertainment tools in the traditional classroom may distract students during the learning process [25]. It seems challenging to pick suitable digital tools to aid teaching without official virtual-tool-guiding guidelines [47,48]. Thus, most teachers prefer to use instant-messaging apps to connect with students after class not during the class. Additionally, some college professors have forbidden students from using electronics in the classroom. Student usage of digital devices in the classroom may be primarily motivated by boredom and distraction in the classroom [49]. In addition, several students have said that using electronic devices in class might distract them, making it difficult for them to keep sustained concentration on their studies [49,50,51]. Because of the lack of comprehensive knowledge and comparison of virtual tools, it is difficult to choose a specific digital tool [47,48].

### Brief Summary of Technology Support Learning before COVID-19

We discovered through surveys that most of the tools used by teachers before the pandemic had the following characteristics. First, most of the virtual tools using were user-friendly and basically used from a smartphone with Internet access. Teachers and students may readily use this before, during, and after class, which is extremely convenient. Furthermore, there is no need to spend extra time learning how to use these virtual tools, because these are simple to use and quick to get started with. From the perspective of a use scenario, these technologies are more primarily employed outside the classroom, as a way to assist students in expanding their learning resources or strengthening communication between teachers and students.

The researcher presented the following findings in view of how technology might enhance teaching and learning pre-pandemic as follows: First, the survey above shows that teachers often use online learning tools as an adjunct to traditional teaching. Few teachers solely relied on virtual tools or online resources pre-pandemic [43]. This is undoubtedly something to do with the risks that come with online education. The online learning environment lacks peer social interaction and instructor supervision, but demands excellent time management from students [52,53,54]. It is difficult for online learning to replicate the advantages of traditional classrooms, such as a sense of presence and social interaction. Thus, before the pandemic, teachers preferred using virtual tools as additional supportive tools to help students learn outside the classroom. MOOC platforms and YouTube webset are examples. Similarly, instant-messaging applications may help teachers stay in contact with their students after class. Chinese teachers, in particular, prefer using WeChat to send notifications and tasks after class [55]. From the perspective of accessibility, MOOC platforms, YouTube video sites and instant-messaging applications offer unlimited access on smartphones or personal computers. One study claimed that about 85% of pupils use smartphones [56]. It is reasonable to suspect that one of the factors influencing teacher decisions to use these virtual tools in learning is the widespread use of cellphones, which has made it easier to use these virtual tools. Additionally, some professors dislike it when students use electronic devices in class. They fear it will divert student attention [57].

Technology does not seem to be fully incorporated into the face-to-face teaching process. Rather, it appears to be used more as additional supportive tools to assist teaching. There is still some trepidation over the broad use of electronic devices in the classroom, it seems that prior to the pandemic, virtual tools were used in approximately the following two circumstances. The first is that virtual tools will be advocated for use in flipped classrooms and blended learning as a means of enhancing classroom materials, e.g., providing a selection of instructional MOOC videos or practice websites for activities specified by students. As a result, the application scenario of virtual tools is appearing more after class. The second is using instant-messaging apps such as WeChat and WhatsApp to stay in touch with students after class. Unlike email, which may take time to reach its intended recipient and is sometimes considered spam, instant-messaging software not only allows for fast message sending and receiving, but also provides a range of long-distance communication options.

## 4. Characteristics of General Online Learning and Teaching That Have Been Used during COVID-19

In March 2020, the World Health Organization declared coronavirus 2 (SARS-CoV-2) to be a worldwide pandemic [58]. Most schools choose to close temporarily to safeguard student and teacher safety. The existing educational system faced a tremendous challenge as a result of school closures. As soon as lockdown was announced, all classroom activities had to be shifted to online platforms so that students could continue their normal study schedules. Hodges identifies these alternative pedagogical practices in COVID-19 as Emerging Remote Teaching [59]. Emerging Remote Teaching (ERT) refers to the temporary shift from a traditional teaching format to a pre-determined alternative teaching format in a health crisis [59]. The pedagogical solutions covered by ERT include a complete online learning program during the lockdown. As of 2002, the term web2 appeared along with several websites that made online transactions and connectivity easy [60]. The web2 tools support the display of multimedia content and interactive applications using two-dimensional screens and the web [61]. Knowledge and information are more readily accessible and delivered with the development of web2 technology. Several web-based user-centric information platforms and applications are widely used in teaching, such as YouTube and blogs [62].

Some virtual tools such as MOOC, WhatsApp, and LMS developed rapidly before this health crisis; however, it is still challenging for teachers and students to use virtual tools expertly in a purely online environment without adequate preparation [63]. A few of these challenges include rationalizing course schedules, learning how to use various virtual tools, and monitoring or evaluating the learning process. Virtual tools have been critical to making online instructions work well during this health crisis. In this study, we divide the pandemic into two different periods based on teacher adaptations to online learning: the emergency transition period and the gradual adaptation period.

### 4.1. Teaching and Learning in the Emergency Transition Period

Social distance and school lockdown in the early period of COVID-19 impacted learning [64]. During lockdown, students had to complete all learning tasks at home via the Internet, and teachers and students wanted connections quickly. In a crisis situation, Synchronous Meeting Tools (SMTs), instant-messaging apps, and live-streaming platforms are preferred as the main instructional tools for online learning. Synchronous Meeting Tools (SMTs) such as Voov Meeting and Zoom are often used in webinars, online meetings, and online lectures. These tools provide high-quality video and audio interconnection between users [65]. Screen-sharing and annotation make the meeting tool suitable for online instruction. A survey at Tsinghua University showed that 97% of teachers chose Voov Meeting software to teach during the COVID-19 pandemic [66]. Due to its accessibility, Voov Meeting was the main platform of choice for teachers in China during COVID-19. Wiranota et al. found that students were happy with Voov Meeting, which was well-suited to assist online learning activities during the pandemic [65]. Similarly, Benazir et al. found that Voov Meeting had a significant positive impact on online learning [67]. However, lacking group discussion was identified as a disincentive to promote deeper communication in online learning [68]. In contrast to Voov Meeting, another synchronous meeting tool, Zoom, has a group discussion feature that helps students and teachers to construct private conversations and group discussions. One study showed that more than half of students felt that the group discussion function in the meeting software helped them communicate comfortably in specific contexts [69]. In addition to using synchronous meeting tools (SMTs) to connect with students, some teachers chose social media platforms with live streaming such as YouTube live streaming or instant-messaging applications such as WhatsApp and Facebook group for video or text communication [70,71,72].

Because of the pandemic, learning activities were instantly relocated online and made available via the use of virtual tools. Synchronous meeting tools enabled students and teachers to communicate in real time outside a traditional classroom at the beginning of the pandemic via an Internet connection. Another major obstacle in pure online learning during COVID-19 was weak social connection because of social isolation. In the traditional classroom, teachers and students preserved close social connection. This social interaction was weakened by the physical distance measure in the pandemic. Apart from the usual knowledge transition, tools were also needed to temporarily moderate the weakened social connection between teachers and students, who were both isolated at home. At that time, instant-message tools that are essential to people’s day-to-day lives started gaining the attention of both teachers and students. Compared with other virtual tools, instant-messaging apps are easy to use and do not require a lot of training. Instant-messaging app with strong accessibility. Through synchronized communication tools, users can log in immediately from their phones once connected to the Internet. This shortens the entire process of sending and replying to messages. Students and teachers may have repaired communication channels temporarily through social media applications, but online chats are not a satisfactory replacement for face-to-face communication. Additionally, live classes seem to be the best method of teaching theoretical subjects [73].

#### Brief Summary of Learning Features in the Emergency Transition Period

The most essential thing for both students and teachers during the emergency transition phase is to return to classes and ensure that they are connected. As a result, the alternatives for students and instructors are technologies that may fulfill basic connection, social interaction and convenience of use. Synchronous meeting tools and instant-messaging apps were included as alternatives. Furthermore, these tools are widely available and adequate to allow the usage of mobile phones or computers based on network connection. Second, they have a user-friendly design and are simple to use, requiring no additional device support. It is also worth mentioning that neither synchronous meeting tools nor instant messaging applications were intended expressly for learning, yet these were widely used in the ERT learning process during the pandemic [74].

The following conclusions were offered in light of how technology may improve teaching and learning during the emergency transition period. According to the results of the above research and survey, most schools adopted a purely online model of teaching and learning during the emergency, and teachers were forced to search for a virtual tool to support purely online learning. A synchronized meeting tool enabled teachers to create an online learning environment that encouraged social interaction during the pandemic, allowing students and teachers to communicate via HD video call in real time. Although previous studies have shown that the quality of teaching using SMTs is lower than that of students in traditional classrooms, it is still an appropriate way to build connections with students quickly during times of health crises [75]. In addition to developing HD video calls, building social interactions with students is another crucial requirement. It makes sense that instant-messaging apps evolved into the primary method for sustaining social interactions given during COVID-19 how commonplace their use became and how significant a role they played in people’s lives. During the pre-pandemic period, there were also examples of summer distance learning using synchronous meeting tools. In addition, with the support of social media, the process of teacher–student interaction and knowledge sharing is enhanced, while student presence in learning has increased [76]. Additionally, social media also in one way ensures that students can receive support and connections from their peers. During the quarantine period of the pandemic, students lost support from their peers, and this isolation was not conducive to learning [77]. With the use of social media and instant-messaging apps, students can connect with classmates and friends, communicate about learning and life difficulties, and help each other through the difficult quarantine period. To a certain extent, social media and the instant messaging app provide positive emotional support to students. The socially weak interactions between teachers and students due to social–physical isolation are gradually alleviated by the real-time interactions supported by social media.

### 4.2. Teaching and Learning in the Gradual Adaptation Period

**Figure 4:**
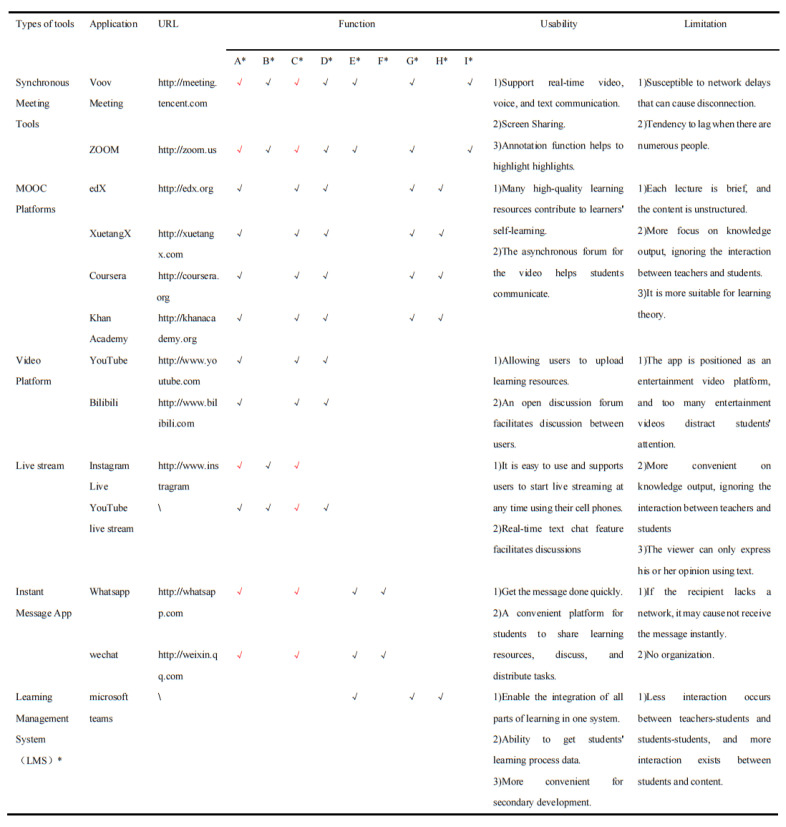
Compare different virtual-tool characters, functions, advantages and limitation; A*: Video and voice presentation: one way interaction in black and bidirectional interaction in red; B*: Real-time interaction; C*: Text-only communication: synchronous interaction in black and asynchronous in reds interaction in red; D*: Screen Sharing; E*: Collaborative Learning; F*: Notice; G*: Test; H*: Learning Process Record; I*: Built-in Recording; Since the specific functions of LMSs are different, we will just discuss their general functions.; LMS*: learning management system is an application that support most learning activities in a platform.

As online learning practice has progressed, teachers have gained an in-depth understanding of the various virtual tools. Teachers are increasingly relying on online virtual tools such as Google Classroom and other cloud-based learning-management system and instant-messaging apps to teaching. Different digital tools often have different functions, and using only one tool does not fully meet the individual needs of students to a certain extent. To maximize the effectiveness of online learning, most teachers tend to use multiple virtual tools simultaneously. Studies have proved that using virtual tools in combination during the pandemic increased online learning efficiency and had a positive influence on learning outcomes [73,78,79]. Figure 4 depicts a horizontal functional comparison of the virtual tools that were frequently used during the pandemic.

According to the literature, several common ways of using virtual tools in combination exist in lockdown, e.g., teaching with MOOCs and synchronous meeting tools in lockdown. It enables students to study at their own speed while communicating with the teacher in real time, resulting in a high-quality experience of distance learning. The research of McClure al. shows that student mastery of concepts and skills is enhanced by providing them with pre-recorded video material. At the same time, synchronous meeting tools (SMTs) provide a platform to help students form a sense of identity in synchronous communication. This combination of self-paced learning and synchronous communication help to increase student self-regulated learning tactics and sense of identity [80].

Apart from that, another typical method adopted by teachers in lockdown was the usage of learning-management system and instant-messaging apps software at the same time. Prior to the COVID-19 outbreak, learning managements system were characterized as an all-in-one learning tool that allowed students to access learning materials, exercises, quizzes, and other learning activities. Learning-management systems make it easier to incorporate instructional resources, papers, and statistics from the student learning process. Learning-management systems were mostly used in conventional face-to-face learning environments in the past. However, the implementation of learning-management systems has been restricted owing to the high cost of development. The occurrence of COVID-19 has rekindled teacher interest in learning-management systems. During the pandemic, teachers and students were physically separated in different places. instant-messaging apps applications such as WhatsApp and WeChat also facilitate communication during lockdown period. When students are using the learning-management system for learning, WhatsApp, WeChat, and other instant-messaging apps may serve as a substitute for face-to-face class contact. This is one of the reasons why instant-messaging apps gained popularity among teachers.

In addition to the feature of using multiple tools in combination, another typical characteristic is that instant-messaging apps became a must-have tool during a lockdown. Teachers and students are clearly limited to their homes, and all learning activities occur online. In this case, it is especially critical to send and receive information instantly during the learning process. Data from a study in Indonesia showed that 94% of the 212 lecturers surveyed chose to use WhatsApp for real-time communication, course notifications, material sharing, and task distributions [81]. Some researchers argue that instant-messaging apps have become necessary in online learning especially in COVID-19 [82]. Instant-messaging apps can support smooth communication between teachers and students. Therefore, more teachers prefer using instant-messaging apps to send notifications during the pandemic. The isolation of physical distance makes it particularly important for teachers to have smooth real-time communication with students. These instant-messaging apps supported immediate notification, such as course reminders, assignment collection, and the scheduling of teaching plans during COVID-19.

#### Brief Summary of Learning Features in the Gradual Adaptation Period

We discovered through surveys that online learning in the gradual adaptation had the following characteristics.Teachers gained specific lessons in online learning environments and began to gradually explore high-quality online instructional design and strategies, which contrasted with the urgency of the pandemic’s early breakout. As opposed to the passive selection at the beginning of the project, teachers gradually and actively investigated how to apply the various tools to different teaching scenarios as they learn about the functions of the different tools. Additionally, using many tools in combination becomes normal. The main criterion for the combination of technologies is to guarantee that the online course runs smoothly while also allowing for social interaction between lecturers and students. Furthermore, tools to sustain social connections are necessary for online learning. Keeping social contact is a critical task for teachers. Thus, teachers prefer to use digital technology tools to maintain the normal instruction process and instant-messaging apps to keep communication flowing.

The following presents how technology may improve teaching and learning during the gradual adaptation period. Unlike in the early days of the pandemic, when teachers were trying to discover the appropriate tools to start online learning, teachers started to explore tool-based approaches for effective online learning. In addition, in this day and age, the classroom is frequently based on a collection of various virtual tools. The teacher progressively masters how to use these tools as well. Given the weak social connection in online learning, teachers frequently use instant-messaging apps to improve social interaction. Despite the transition from an emergency transition period to a gradual adaptation period, many instructors continue to use non-education specialized online platforms such as Zoom, VooV meeting, YouTube etc. This is an enthralling phenomenon.

## 5. Challenges with Online Learning during the Pandemic

People moved all activities to the Internet because of the outbreak of COVID-19. Especially in education, all learning activities in the traditional classroom were moved to the Internet. Compulsory online learning practices allowed teachers to make a more comprehensive view to digital technology [83]. The new normal was first used in the economic field. It refered to the return of the industrial economy to its usual level after a recession [84]. Students slowly returned to school as the pandemic began to calm down. A growing number of researchers have begun to rethink the experiences of lockdown. Researchers have started to explore the future direction of digital transformation in education and the new normal of education in the post-pandemic era. During the pandemic, more teachers realized that digital technologies may deliver high-quality learning services for students. However, online learning during the pandemic still met many challenges because people were not fully prepared to adapt when COVID-19 came.

First, online classes were occasionally interrupted because of poor Internet access [85]. Students struggled due to the inconsistent Internet connections. Higher network latency and slow buffering speeds seriously harmed learning experiences [86]. Another challenge in pure online learning is the low-quality interaction in online classes [87,88]. By surveying 840 medical students’ feelings about online learning experiences during the pandemic, Bkaczek et al. discovered that most students felt lonely in online learning due to a lack of contact with teachers and peers [89]. Furthermore, the interaction supported by virtual tools always happened between students and content [90,91]. Students and teachers want a well-connected experience in online learning. As early as 2004, experts proposed that online learning might contribute to a feeling of isolation and that this loneliness may lead to the failure of online education [92,93]. However, with the rapid development of information communication and technology in a decade, such a challenge remains unresolved. Before the outbreak, information technology advanced quickly, with more virtual-tool choices accessible to teachers and students. However, the disadvantages of online education that have previously been investigated seem to be present during lockdown. The irrefutable fact is that the traditional face-to-face classroom is still the main form of teaching, despite the rapid development of digital tools. The third challenge is that the combined use of multiple tools adds to students’ cognitive load [94]. Working memory is consumed by attending to many physically dispersed sources of important information at the same time, resulting in an increased cognitive load [94]. In fact, using multiple digital tools in combination was the norm in online learning during the pandemic. To keep up with all the notifications, video calls, assignment submissions and evaluations that come with online learning, it is necessary to use many applications simultaneously. However, this combination of multiple virtual tools in online learning is still not a sustainable approach. On the one hand, learning with multiple tools is cognitively burdensome for those students who spend much time switching different tools. Students must balance their attention over multiple channels of information presentation, and switching back and forth between several channels of information processing at the same time. Prolonged multitasking leads students to have a high cognitive load [94]. There are even studies that show that students seem to benefit less from this type of education [95]. On the other hand, learning in multiple apps scatters student learning process data across different platforms, which is not conducive to effectively collecting and using student learning process data. For both teachers and students, it takes a lot of extra time to learn how to make full use of these various tools. Because of these emerging challenges, most students still choose to return to traditional study rather than online learning when the school reopens. Overlaying different virtual tools in an online learning scenario is also not the best solution for the post-pandemic era or another unforeseeable health crisis. Using multiple tools simultaneously to meet various learning needs is not the way forward for the digital transformation of education. After a period of practice with online education, universities are gradually becoming stuck with a difficult decision about whether to reinstate face-to-face classes [96]. The poorly received online learning effect [97], the overlapping use of multiple tools [98], network connectivity and security issues [99] have become one of the many challenges of online learning during the pandemic. For these reasons, after the outbreak has stabilized, some studies say that returning to school for classes, rather than continuing distance learning via the Internet, is the best option [100,101].

## 6. Conclusions

The outbreak of COVID-19 has caused tremendous damage to society, public health and education. With no other choices, most schools were forced to convert the offline face-to-face classroom model to an online one. Even though the pandemic has disrupted people’s lives, it has also changed their habits. With the pandemic gradually stabilizing, people began to seek lessons from practices already in place. It was widely accepted that synchronized meeting software would remain an alternative for emergencies after the outbreak.

We mentioned in Section 3 that digital transformation in education involves finding appropriate ways to apply digital technologies The highest vision of digital transformation is to transform traditional face-to-face instruction into online and blended instruction.

The acceptance of new technologies is one of the most important factors For technological success for technological success [102]. It usually takes some time for technology to be adopted and applied in a new environment in normal circumstances [103]. Education is not an exception to this situation.

Research has viewed COVID-19 as an opportunity for gaining experience in the future digital transformation of education, as the great spread of COVID-19 has resulted in people using digital technology more frequently in everyday life [104]. It is clear that the COVID-19 pandemic has accelerated the rate at which technology is becoming a part of everyday life and education [105]. However, during the pandemic, students and faculty seemed to simply transfer material from traditional courses to the web [106]. It is clear that a truly transformative vision of digital learning remains a long way off, despite the large-scale use of technology-assisted learning environments during the COVID-19 pandemic. Using virtual tools in the learning process is only one aspect of digital transformation [107].

As we explore digital transformation, we cannot deny that existing virtual tools to support education are very valuable, especially the experience gained through pandemic-era online education. As a matter of fact, this is our point of view as well. Although not entirely good, the COVID-19 period has yielded some experience for the digital transformation of education through online learning. During this pandemic, online tools based on web2 were used to support most teaching activities. This seems to be similar to what was once mentioned about making digital technology fully integrated into teaching and learning. Accordingly, this study concludes that it is necessary to summarize and analyze the characteristics, advantages, and challenges of virtual-tool-supported learning, especially during pre-pandemic and pandemic situations. The accumulation of this large-scale experience of online learning has prepared for the digital transformation of education in the foreseeable future. Additionally, this will prepare the field of education to be more resilient in the event of future unforeseeable health risks.

Based on the above analysis, this study examines nearly 20 years of research on virtual tools for assisted learning and finds that the method of technical support education was very different from what it was in the pre-pandemic era and during the pandemic. The study found that virtual tools mainly served as a supplement to traditional face-to-face classes in the pre-pandemic era. Most teachers were still at a preliminary stage of understanding most virtual tools at that time. Learning activities must be moved to Internet platforms due to the lockdown. This experience allowed teachers to affirm the critical role of the virtual tool in learning, especially during a particular period, i.e., health crises. However, some practical challenges affected the quality of online learning that existed during the lockdown. The challenges that appear in COVID-19 are discussed. Most virtual tools only meet basic needs in learning. First, existing virtual tools were insufficient to support the construction of more immersive learning scenarios. Second, there was difficulty achieving deep interaction between teachers and teachers. Finally, there is a lack of a so-called all-in-one tool. There actually have been some bad experiences with online virtual learning tools, and these tools are underused. An all-in-one tool that can make up for the above shortcomings could provide a new approach to thinking about these problems in the future compared to web2 virtual tools such as those used during the pandemic, especially for the digital transformation.

Additionally, the study aims to provide a reference for teachers or educational researchers, particularly for reducing the health crisis impact on life and unforeseeable learning scenarios. It is hoped that the analysis above will shed light on the role and function of online learning in the aftermath of the recent global health crisis. In the end, we hope to see a new reality in which technology meets society to provide a more engaging and adaptive science conference experience and allows for a more adaptable and dynamic use of content, tailored to each learner’s needs. 

## Figures and Tables

**Table 1:** The keywords search string.

Database	Keywords
Web of Science	TS = ((“online learning” OR “online teaching” OR “distance learning” OR “distance education” OR “e-learning”) and (“technology support learning” OR “technology-assisted learning” ) and (“online learning platform” OR “online learning tool” OR “virtual tools”) and (“learning model” OR “learning method” and (“effect” OR “impact” OR “influence”) and (“LMS” OR “tool” OR) and (“COVID-19” OR “pandemic” OR “health crises” OR “health crisis”))

**Table 2:** The specific criteria of inclusion and exclusion.

Criteria	Eligibility	Exclusion
Document Type	Article	proceedings paper, book chapters, data paper, editorial materials, early access, review article
Language	English	Non-English
Time Line	1 January 2008–1 May 2022	<2008

**Table 3:** Specific inclusion and exclusion criteria based on study aim and theme.

Inclusion Criteria	Exclusion Criteria
Studies should be relevant to the technology-supported teaching and learning education field.	Studies that describe technological advances in the non-educational field.
Studies should describe specific instructional approaches supported by technology.	Studies used only questionnaires to investigate students’ or teachers’ perceptions of teaching styles.
Studies should include the experiences of teachers or students.	Studies that did not include the real experiences of students or teachers.
Studies should include a description of the student’s learning outcomes.	Studies that do not address learning outcomes.
Studies related to the pandemic should have described how technology can support online education.	Studies simply describe the impact and challenges of the outbreak on education.
Full text available	

## Data Availability

Not applicable.
